# Internet addiction disorder: When technology becomes a problem

**DOI:** 10.1192/j.eurpsy.2021.1703

**Published:** 2021-08-13

**Authors:** M. Trigo

**Affiliations:** Psychiatry, Centro Hospitalar Universitário do Algarve, Faro, Portugal

**Keywords:** Internet addiction, social media, Addictive disorders, technology addiction

## Abstract

**Introduction:**

Internet addiction disorder (IAD) is the compulsive and problematic use of the internet, resulting in significant functional impairment in several life domains. This happens when an individual engages in online activities disregarding daily responsibilities or other interests, and not realizing its negative consequences. Although not officially recognized as a disorder in the Diagnostic and Statistical Manual of Mental Disorders, Fifth Edition (DSM-V), the relationships between digital media use and mental health has been under debate and discussion amongst experts due to presenting some features of excessive use, withdrawal phenomena, tolerance, and negative repercussions typical of many substance abuse disorders.

**Objectives:**

To present an overview of theoretical considerations on IAD and its eventual inclusion in the next version of the DSM.

**Methods:**

Review of the most recent literature regarding internet addiction disorder. The research was carried out through the PubMed, MedLine, SpringerLink and LILACS databases, using the terms “internet addiction”, “addiction disorders” and “social media”, until December 2020.

**Results:**

There is controversy around the diagnosis of internet addiction, including whether it is a unique clinical entity or a manifestation of other underlying psychiatric disorders, raising complex questions of causality. Since there are no standardized definition, there is lack of evidence-based recommendations to its approach.

**Conclusions:**

Research suggests that some individuals dealing with internet addiction are at significant risk, therefore merit professional care. Further research is needed, with carefully controlled studies, emphasizing incapacity, prognosis and response to treatment, in order to consider internet addiction as a disease, and include it in DSM’s next edition.

**Disclosure:**

No significant relationships.

